# Macrosomie, dystocie des épaules et élongation du plexus brachial: quelle est la place de la césarienne?

**DOI:** 10.11604/pamj.2016.25.217.10050

**Published:** 2016-12-06

**Authors:** Mehdi Kehila, Sadok Derouich, Omar Touhami, Sirine Belghith, Hassine Saber Abouda, Mariem Cheour, Mohamed Badis Chanoufi

**Affiliations:** 1 Service C de Gynécologie-Obstétrique, Centre de Maternité et de Néonatologie de Tunis, Université Tunis El Manar, Tunisie; 2 Service A de Gynécologie-Obstétrique, Centre Hospitalier Universitaire, Charles Nicole, Université Tunis El Manar, Tunisie; 3 Service de Néonatologie, Centre de Maternité et de Néonatologie de Tunis, Université Tunis El Manar, Tunisie

**Keywords:** Macrosomie, dystocie des épaules, plexus brachial, césarienne, Macrosomia, shoulder dystocia, brachial plexus, caesarean section

## Abstract

L'accouchement d'un fœtus macrosome est un accouchement à haut risque de complications maternofoetales. La dystocie des épaules reste la complication fœtale la plus redoutée, amenant au recours, parfois démesuré, à la césarienne. L'objectif de ce travail est d'évaluer l'intérêt de cette césarienne préventive. Il s'agit d'une étude rétrospective à propos de 400 accouchements de fœtus macrosomes survenus entre Février 2010 et Décembre 2012. Nous avons aussi identifié les cas de nouveau-nés ayant eu une dystocie des épaules pendant l'année 2012 ainsi que leur poids de naissance respectifs. Les fœtus macrosomes ont pesé entre 4000g et 4500g dans 86,25% des cas et entre 4500 et 5000 dans 12,25% des cas. L'accouchement était par voie basse dans 68% des cas. Parmi les 400 accouchements de macrosomes, 9 cas de dystocie des épaules ont été enregistrés (2,25%). Tous ces cas sont survenus lors d'accouchements par voie basse. Le risque de dystocie des épaules lors d'un accouchement par voie basse a augmenté de façon significative avec l'augmentation du poids à la naissance (p<10-4). Le risque d'élongation du plexus brachial était de 11 pour mille accouchements de macrosomes par voie basse. Ce risque n'était pas corrélé au poids de naissance (p=0,38). Le risque de séquelles post-traumatiques était de 0,71%. La dystocie des épaules a concerné un fœtus macrosome dans 58 % des cas. La dystocie des épaules n'est pas une complication exclusivement associée à la macrosomie. Le dépistage des accouchements à risque et le renforcement de la formation des obstétriciens sur les manœuvres à réaliser en cas de dystocie des épaules semblent être les meilleurs moyens pour éviter ses complications.

## Abstract (en)

The delivery of a macrosomic infant is associated with a higher risk for maternofoetal complications. Shoulder dystocia is the most feared fetal complication, leading sometimes to a disproportionate use of caesarean section. This study aims to evaluate the interest of preventive caesarean section. We conducted a retrospective study of 400 macrosomic births between February 2010 and December 2012. We also identified cases of infants with shoulder dystocia occurred in 2012 as well as their respective birthweight. Macrosomic infants weighed between 4000g and 4500g in 86.25% of cases and between 4500 and 5000 in 12.25% of cases. Vaginal delivery was performed in 68% of cases. Out of 400 macrosomic births, 9 cases with shoulder dystocia were recorded (2.25%). All of these cases occurred during vaginal delivery. The risk for shoulder dystocia invaginal delivery has increased significantly with the increase in birth weight (p <10-4). The risk for elongation of the brachial plexus was 11 per thousand vaginal deliveries of macrosomic infants. This risk was not correlated with birthweight (p = 0.38). The risk for post-traumatic sequelae was 0.71%. Shoulder dystocia affectd macrosoic infants in 58% of cases. Shoulder dystocia is not a complication exclusively associated with macrosomia. Screening for risky deliveries and increasing training of obstetricians on maneuvers in shoulder dystocia seem to be the best way to avoid complications.

## Introduction

La définition de la macrosomie fœtale est variable d'un auteur à un autre. Ainsi, certains la définissent par un poids fœtal supérieur au 90^ème^ percentile par rapport à l'âge gestationnel et d'autres retiennent comme définition un poids fœtal supérieur à 4000g [1]. Quel que soit la définition adoptée, l'accouchement d'un macrosome est un accouchement à haut risque materno-fœtal. Ainsi, les risques maternels sont dominés par l'hémorragie du post-partum et les déchirures obstétricales [2]. Quant aux risques fœtaux: c'est l'asphyxie néonatale et les lésions traumatiques secondaires essentiellement à la dystocie des épaules qui sont le plus à craindre. La macrosomie est sans nul doute le facteur de risque principal de la dystocie des épaules : en effet, le risque est multiplié par 16 en cas de poids de naissance supérieur à 4000g [3]. Cette complication reste la hantise majeure de tout obstétricien lors de l'accouchement d'un macrosome l'amenant parfois à des indications larges de césarienne préventive. Certes, cette attitude diminue le risque de dystocie des épaules mais elle augmente le taux de complications maternelles propres à la césarienne (hémorragie du post-partum, placenta accreta, décès maternels…) [4]. Le but de notre travail a été donc d'évaluer le véritable risque de dystocie des épaules et de ses complications en cas d'accouchement de macrosome ainsi que l'intérêt de la césarienne préventive dans cette situation.

## Méthodes

Notre étude est composée de 2 parties.

### Première partie

Il s'agit d'une étude rétrospective réalisée au service C du Centre de Maternité et de Néonatologie de Tunis (CMNT) à propos de 400 accouchements de fœtus macrosomes entre Février 2010 et Décembre 2012. Le CMNT est une maternité universitaire de niveau III comprenant 3 services de gynécologie-obstétrique et réalisant une moyenne de 15 000 accouchements par an. Nous avons retenu dans cette étude come définition de la macrosomie un poids de naissance supérieur ou égal à 4000 g [3]. Ont été inclus dans l'étude : tous les accouchements à terme d'un nouveau-né pesant 4000 g ou plus et ce quelque soit la voie d'accouchement. Ont été exclus de l'étude les cas de mort fœtale in utéro. Dans notre maternité, l'épreuve de travail est acceptée quand le poids fœtal estimé (cliniquement ou à l'échographie) le jour de l'entrée en travail est inférieur à 4500g en l'absence de diabète et à 4250g en cas de diabète chez la mère. En cas de suspicion clinique ou échographique de macrosomie, une estimation échographique du poids fœtal est refaite par un sénior. En cas d'utérus cicatriciel la macrosomie ne contre-indique pas à elle seule l'épreuve utérine. La décision prend plutôt en considération les conditions obstétricales, la descente de la tête fœtale ainsi que l'accomodation foeto-pelvienne. Le résident et le sénior de garde doivent être avisés pour être présents lors de l'accouchement afin de pouvoir réaliser les manœuvres à temps si besoin. A noter que les gardes en Tunisie ne sont pas séniorisées. Deux résidents sont quotidiennement de garde sur place dans notre service, le sénior est de garde à domicile, il est appelé en cas besoin. Tous nos résidents pendant la période d'étude avaient déjà effectué au minimum 3 ans de résidence en gynécologie-obstétrique. En cas de dystocie des épaules, la conduite est standardisée: La première manœuvre à réaliser est le manouvre de Mac-Roberts suivie de la manœuvre de Wood inversée; la manœuvre de Jacquemier est réalisée en dernier recours. Les cas de macrosomie ont été identifiés grâce à un système de codage et le recueil des données a été effectué à partir des dossiers d'hospitalisation et d'accouchement. Pour chaque patiente, nous avons recueilli les données anamnestiques, le déroulement et le mode d'accouchement. Pour chaque nouveau né, nous avons noté le poids à la naissance, le score d'Apgar ainsi que les éventuelles complications néonatales.

### Deuxième partie

A partir du cahier d'accouchements du CMNT (registre commun pour les 3 services) et du registre du service de néonatologie du CMNT, nous avons relevé pour l'année 2012: tous les cas d'élongation du plexus brachial survenus dans les 3 services du CMNT, la voie d'accouchement et les poids de naissance des nouveaux nés ayant présenté cette complication. L'étude statistique a été faite avec le logiciel SPSS 20 (SPSS Inc., Chicago, IL). La comparaison entre les groupes a été faite par le Test Khi 2 pour les variables qualitatives et un p < 0,05 était considéré comme significatif.

## Résultats

Pendant la période d'étude, 5624 patientes ont accouché dans le service C. Notre étude a concerné 400 macrosomes, soit 7,11% du total des accouchements. La répartition des poids de naissance est représentée dans le Tableau 1. La moyenne d'âge des parturientes était de 30,9 ans [extrêmes 19 - 44]; 22,5% étaient des primipares, 19% étaient diabétiques et 17,5% avaient une prééclampsie. L'accouchement s'est fait par césarienne dans 32% des cas. Vingt et un accouchements (5,25%) ont nécessité une extraction instrumentale (par Forceps dans tous les cas). Trois décès néonataux ont été recensés (0.7%): deux dans le cadre d´un syndrome polymalformatif et un secondaire à une hypoglycémie. Vingt et un cas d´asphyxie néonatale (Score d´Apgar <7 à 5 minutes) ont été noté (5,25%) et 2,75% des nouveaux nés ont présenté une détresse respiratoire néonatale. Le taux d´admission des nouveaux nés en service de néonatologie était de 8,75% avec une durée de séjour moyen de 2,1 jours.

**Tableau 1:** Répartition des poids à la naissance des fœtus macrosomes

Poids de naissance	Nombre de cas	Pourcentage
4000-4500	345	86,25%
4500-5000	49	12,25%
>5000	6	1,5%

Parmi les 400 accouchements, 9 cas de dystocie des épaules ont été recensées. Ainsi, le risque de dystocie des épaules était de 2,25% en cas de macrosomie. Tous ces cas sont survenus lors d'accouchements par voie basse. Dans notre série le risque de dystocie des épaules était donc de 3,23% en cas d'accouchement d'un macrosome par voie basse et nul en cas d'un accouchement d'un macrosome par césarienne. Cette dystocie a été réduite par la manoeuvre de MacRoberts dans 6 cas, la manœuvre de Wood inversée dans 2 cas et la manœuvre de Jacquemier dans un cas. Ces manœuvres ont été réalisées par des résidents pendant la garde dans 8 cas et une manœuvre de Wood inversée a été réalisée par un obstétricien sénior. La répartition de ces cas de dystocie selon le poids de naissance est présentée dans le Tableau 2. Le risque de dystocie des épaules lors d'un accouchement par voie basse a augmenté de façon significative avec l'augmentation du poids à la naissance (p<0,0001). Il est passé de 2% lorsque le poids fœtal était entre 4000 et 4500g et à 100% lorsque le poids a dépassé 5000g (Tableau 2)

**Tableau 2:** Répartition des cas de dystocie des épaules en fonction du poids à la naissance

Poids de naissance (g)	Nombre d’accouchements par voie basse	Nombre de dystocies des épaules	Taux de dystocie des épaules	p
4000-4500	245	5	2,04%	p<0,0001
4500-5000	26	3	11,6%	
>5000	1	1	100%	

Concernant les complications traumatiques secondaires à la dystocie des épaules, on a relevé: a) un cas de fracture de la clavicule avec élongation du plexus brachial (EPB) chez un fœtus ayant pesé 4900 g. L'évolution était favorable avec consolidation de la clavicule et une régression totale de la paralysie brachiale après 9 mois de kinésithérapie; b) deux cas d'EPB chez des nouveau-nés des poids de naissance respectifs de 4150g et de 4400g. L'évolution était marquée par la persistance d'une parésie du membre supérieur dans ces 2 cas.

Ainsi, le taux d'EPB dans notre série était de 7 pour mille accouchements de fœtus macrosomes. Ce risque était donc de 11 élongations de plexus brachial pour mille accouchements de macrosomes par voie basse. En cas de dystocie des épaules, le risque d'EPB était de 33.3%. Ce risque n'était pas statistiquement corrélé au poids de naissance (p=0,38). Le risque de séquelles post-traumatiques était de 0,71% (soit 1/141). En d'autres termes, la réalisation de 141 césariennes pour macrosomie aurait permis d'éviter un seul cas de séquelle faisant suite à une dystocie des épaules. Aucun cas d'asphyxie (Apgar <7 à 5min) ou de décès néonatal n'a été noté suite à une dystocie des épaules.

Pendant l'année 2012, 14950 accouchements ont eu lieu au Centre de Maternité de Tunis et 12 cas d'élongation du plexus brachial sont survenus; tous suite à un accouchement par voie basse. Le risque d'EPB était statistiquement associé à l'accouchement par voie basse (p ≤10^-3^; IC95% [0.03; 0.13]). L'incidence de l'EPB était donc de 0.8 pour mille accouchements. Le taux de césarienne pendant cette période était de 46%. Ce taux élevé de césariennes est en partie expliqué par le fait que le CMNT est la maternité de référence de la Tunisie qui draine la majorité des grossesses à risque du pays. L'incidence de l'EPB était donc de 1.7 pour mille accouchements par voie basse. L'EPB a intéressé 7 fœtus macrosomes (58%) et 5 dont le poids de naissance était < 4000 g [extrêmes: 3500g-3900g].

## Discussion

La dystocie des épaules est définie par l'absence de dégagement des épaules du fœtus après expulsion de la tête, rendant nécessaire le recours à des manœuvres obstétricales autres que la traction douce de la tête ou la manœuvre de restitution [5]. Elle survient dans 0,5 à 1% des accouchements par voie basse [5]. Par ailleurs, des cas de dystocie des épaules ont été rapportés suite à un accouchement par césarienne qui n'élimine donc pas totalement ce risque [6]. Il s'agit d'une complication imprévisible mais plusieurs facteurs de risque sont identifiés tel que: l'antécédent de dystocie des épaules, la macrosomie fœtale, le diabète gestationnel, l'obésité maternelle, le sexe fœtal masculin ainsi que l'extraction instrumentale [3, 6–8]. Le risque de dystocie des épaules est multiplié par 16 à 24 fois si le poids de naissance est supérieur à 4000g [3, 7]. Ainsi, la macrosomie fœtale reste le facteur de risque majeur de dystocie des épaules mais non le seul puisque la dystocie des épaules survient chez des fœtus ayant un poids de naissance < 4000g dans 38% à 88% des cas [7, 8]. Le risque de dystocie des épaules dans notre série était de 2,25% en cas de macrosomie et de 3,23% en cas d'accouchement d'un macrosome par voie basse.

Par ailleurs, le risque dystocie des épaules en cas de macrosomie augmente avec le poids de naissance. Ainsi, le risque est multiplié par 6,2 en cas poids de naissance entre 4000 et 4500g [9] alors qu'il est multiplié par 22,7 si le poids de naissance dépasse 5000g [10]. Cette notion a été retrouvée dans notre série (Tableau 1) avec une différence significative entre les différentes catégories de poids de naissance. Toutefois, les complications fœtales de la dystocie et ses séquelles à long terme importent plus que l'évènement en lui-même. Ainsi, la dystocie des épaules peut être à l'origine d'une asphyxie néonatale, d'une élongation du plexus brachial ou d'une fracture de l'humérus ou de la clavicule avec possibilités de séquelles à moyen et à long terme [11]. Le taux cumulé de ces complications fœtales en cas de dystocie des épaules varie entre 22% à 41% [11]. Quant au taux d'asphyxie néonatale en cas de dystocie des épaules (définie par un score d'Apgar inférieur à 7 à 5 minutes), il peut atteindre 7% [12]. Elle est le plus souvent secondaire à un retard de prise en charge de l'accident [12]. Cette complication n'a pas été rapportée dans notre série. Concernant la fracture de la clavicule, son taux est multiplié par 35 en cas de dystocie des épaules [13] mais elle peut survenir même en cas de césarienne et en absence de macrosomie [14]. Son évolution est généralement favorable et sans séquelles [15]. Un seul cas de fracture de la clavicule a été rapporté dans notre série avec une évolution favorable.

Ainsi, la crainte de la dystocie des épaules ne semble pas justifier la pratique systématique d'une césarienne en cas de suspicion de macrosomie et ce pour plusieurs raisons: a) l'estimation échographique du poids de naissance fœtale est loin d'être précise surtout pour les poids de naissance élevés [16]; b) même elle diminue le risque, la césarienne n'élimine pas totalement l'élongation du plexus brachial (1% de risque en cas de césarienne) [6]; c) la survenue de cette complication est imprévisible et multifactorielle et peut survenir même chez des fœtus eutrophiqes [17]. En effet, 50 à 75% des dystocies des épaules surviennent en l'absence de facteur de risque[5]; d) le taux de complications traumatiques et de séquelles fœtales secondaires à cet accident est très faible [13]; e) dans notre étude l'EPB est survenue chez des fœtus eutrophiques dans 42% des cas donc le fait de détecter tous les fœtus macrosomes et leur réaliser des césariennes préventives ne va pas prévenir cette complication; f) beaucoup de césariennes inutiles doivent être réalisées pour éviter un cas d'EPB. Dans notre étude, ce nombre était de 141 césariennes pour macrosomie pour éviter une EPB. Rose et al. estiment qu'en cas de poids fœtal estimé supérieur à 4000, il faut pratiquer 2345 césariennes supplémentaires pour éviter un cas de lésion permanente du plexus brachial. Ceci serait à l'origine d'un décès maternel attribuable à la césarienne pour chaque 3,2 cas évités [18]. Par ailleurs, il a été démontré que le risque de décès maternel est presque trois fois plus en cas d'accouchement par césarienne par à un accouchement par les voies naturelles (OR 2.87, 95% CI 1.63-5.06) [4]; g) la bonne maîtrise des manœuvres obstétricales à réaliser lors d'une dystocie des épaules permet de limiter le taux de complications traumatiques et de séquelles fœtales secondaires. Ceci a été établi dans deux revues de la littérature récentes qui ont montré l'intérêt de la simulation dans la prise en charge de la dystocie des épaules [19, 20].

## Conclusion

Etant donné l'augmentation de risque de dystocie des épaules en fonction du poids de naissance, il serait probablement sage de fixer des limites de poids fœtal au dessus desquelles la césarienne sera la voie d'accouchement privilégiée. Cette limite devrait idéalement être réfléchie au sein de chaque service obstétrical en tenant compte de son potentiel humain et de l'expérience des obstétriciens présents en cas de survenu d'une dystocie des épaules. Cette limite de poids choisie doit prendre en compte aussi l'augmentation du taux de césariennes et de ces complications. Par ailleurs, on doit être bien conscient que ces césariennes itératives, certes, vont diminuer le taux des dystocies des épaules mais celles-ci vont tout de même survenir chez des fœtus eutrophiques ou macrosomes non dépistés. La formation des obstétriciens pour la maitrise des manœuvres à exécuter en cas de dystocie des épaules nous semble le moyen le plus fiable pour diminuer de façon générale le taux des EPB. Cette formation devrait être réalisée de façon régulière sur des mannequins de simulation que doivent se procurer les centres pédagogiques de chaque faculté de médecine ou idéalement être disponibles dans chaque maternité.

### Etat des connaissances actuelle sur le sujet

- Le risque de dystocie des épaules est augmenté en cas macrosomie.

### Contribution de notre étude à la connaissance

- Le risque d'élongation du plexus brachial n'est pas corrélé au poids fœtal;
- Ce risque semble plutôt être en rapport avec les manœuvres réalisées en cas de dystocie des épaules.
